# Understanding wildlife crime in China: Socio-demographic profiling and motivation of offenders

**DOI:** 10.1371/journal.pone.0246081

**Published:** 2021-01-28

**Authors:** Mei-Ling Shao, Chris Newman, Christina D. Buesching, David W. Macdonald, Zhao-Min Zhou

**Affiliations:** 1 Key Laboratory of Southwest China Wildlife Resources Conservation (Ministry of Education), China West Normal University, Nanchong, China; 2 Wildlife Conservation Research Unit, The Recanati-Kaplan Centre, Department of Zoology, University of Oxford, Oxford, United Kingdom; 3 Cook’s Lake Farming Forestry and Wildlife Inc (Ecological Consultancy), Queens County, Nova Scotia, Canada; 4 Department of Biology, Irving K. Barber School of Arts & Sciences Unit 2, University of British Columbia, Okanagen, Canada; Universitat Autònoma de Barcelona, SPAIN

## Abstract

Wildlife crime presents a growing threat to the integrity of ecological communities. While campaigns have raised consumer awareness, little is known about the socio-demographic profile of wildlife offenders, or how to intervene. Using data from China Judgements Online (2014–2018), we documented 4,735 cases, involving 7,244 offenders who smuggled, hunted, transported, sold and/or purchased protected species in contravention of China’s Criminal Law. Offenders were predominantly men (93.0% of 7,143 offenders), aged 30–44 (43.9% of 4,699), agricultural workers (48.4% of 3,960), with less schooling (78.6% of 4,699 < senior secondary school). Socio-economic profiles related to crime seriousness, the type of illegal activity, motivation and taxon involved. These generalizations reveal scope to tailor specific intervention and mitigation approaches to offender profiles, through public information campaigns, proactive incentives opposed by punitive disincentives, and provision of alternative incomes.

## Introduction

Criminology has established that individual demographic characteristics (i.e., offender profile: age, sex, education level, occupation) relate to propensity for delinquency, where consistently young people, especially men, tend to become offenders [1]. Attempts to address the illegal wildlife trade [2,3] have typically focused on consumers, particularly tackling demand through education campaigns [4–6]; else face the disincentive of prosecution and punishment [7]. However, much also remains to be learned from investigating supply chains and understanding offender profiles and motivations [5]. For instance, a recent review by Wilson & Borrato [8] highlights that the ‘tough on wildlife crime’ approach tends to be ineffective, or even counterproductive in reducing offending and recidivism.

To reduce propensity to offend effectively, constituencies susceptible to wildlife crime must be identified, profiled and their motivation understood [5,9–11]. When wildlife crime is ‘need-based’, it is vital to provide alternative incomes; when crime is ‘greed-based’, punitive measures may be appropriate [12,13]. While there are arguments to reform IWT legislation [14], any decision to transgress the law constitutes a criminal action.

Types of offenses vary in sophistication, motive and seriousness, from casual local poaching by individuals through to organized crime cartels trading ivory internationally, alongside smuggling of drugs, counterfeit goods, weapons, etc. [7,15,16]. Who owns wildlife can also have a tacit effect on how individuals perceive their rights to exploit or manage it [17]. For instance, in the United States [18], as in China, wild animals are federal or state property, whereas in the UK [19] and South Africa [20] they are extensively the legal property of private landowners. Like other property crimes [21], categories of wildlife crime may involve different offender typologies, according to sex biases [22], age-crime curves [1], education levels [23], socio-economic backgrounds [13], and previous criminal history (potential reward vs severity of sentencing) [24].

Wildlife crimes are often considered to be ‘victimless’ [25]–a perception that ignores the intrinsic value attributed to animals under a non-anthropocentrist ethic [26], and which in practical terms regards biodiversity loss and animal suffering as too intangible to trigger moral restraint (vs theft, assault, etc). Offenders may take authority to commit wildlife crimes from religion or heritage, including formerly legal activities [27], such as traditional medicine [28] or hunting [29]. Hunting can be encouraged by concepts such as the ‘fearless male’ [30,31]. Sollund [32] reports that 89.1% of wildlife crime in Norway is committed by men, motivated by hegemonic masculinity. This may also take the form of the ‘male provider’, trying to feed his family, or protect crops from wildlife [14].

In overview, Nurse [14] summarized 5 underlying motives to offend: profit/ commercial gain; thrill/ sport; necessity for obtaining food or protecting livestock/ crops; antipathy towards governmental and law enforcement bodies; and traditional/ cultural reasons. Ignorance of the law is sometimes a further justification [33], and sometimes laws are unclear [34]. These motivations offer potential avenues through which wildlife offences, especially crimes of opportunity committed by men, might be redressed: for instance, involving public information campaigns, proactive incentives opposed by punitive disincentives, and provision of alternative incomes.

Here, we focus on China, where any convicted smuggler/trader/hunter could face up to 15 years fixed-term imprisonment accompanied by fines and/or the confiscation of property [35]. The Chinese government and international enforcement agencies are engaged in substantial efforts to reduce animal trafficking [2], where Southwest China has historically been a major wildlife outflow region [36], especially within and around biodiversity hotspots where local economies tend to be under-developed [37], comprised by a large, poor, rural population [38]. In contrast, wildlife influx into China is mostly destined for major economic centers away from Southwest China, where strong demand for ivory [39], rhino horn [40] and pets [41,42] has been linked to an increase in wealth [41,43].

The reform of China’s judicial system from July 2014, offered an unprecedented opportunity to collect high-quality data on criminal prosecutions at the national level. In accordance with China’s Supreme People’s Court, since 1 January 2014, judgement records must be published on China Judgements Online (http://wenshu.court.gov.cn/) within seven days of adjudication, and cannot be amended, replaced or withdrawn without court authority. We used this resource to collate reports pertaining to illegal hunting, trafficking and/or smuggling of species and/or any products derived thereof protected by China’s List of Fauna under Special State Protection (LFSSP), and/or any non-native species listed in the Convention on International Trade in Endangered Species of Wild Fauna and Flora (CITES) Appendix I, II (unless specifically permitted, e.g. for research, domestication or exhibition); all prosecuted under China’s Wild Animal Conservation Law (WACL) and Criminal Law (CL) [35]. Offender identities were anonymized in court documents, but included age, sex, education level and occupation in relation to the type of illegal activity, crime seriousness and wildlife taxon involved. Additionally, in many instances court documents and defendant statements included in case reports allowed us to ascertain offender motivation. From these data, we established specific offender profiles, and then identified targeted measures aimed at addressing this criminality.

## Methods

### Data source

Author M.L.S. searched the China Judgements Online archived between 1 January 2014 and 31 December 2018 for documents containing the term ‘wild animal’ (in Chinese), and then screened these for eligibility according to the following criteria:

- The case involved a protected wild animal species.
- Full text could be accessed.
- A consistent case number was assigned across any retrial documents.
- Each perpetrator was described individually in multiple-offender cases.

Offenders had various occupations, which we categorized into agricultural workers (a collective term applied to include ‘peasants’, ‘fishermen’ and ‘herdsmen’), non-agricultural workers and unemployed. These three categories reflect skill sets required: hunting (tracking, shooting, setting traps), smuggling (foreign language ability, international contacts, knowing foreign laws) and domestic trafficking. In addition, agricultural workers have a lower hunting opportunity cost [44], while the unemployed have free time to devote to illicit activities [45]. We ascertained offender motivations from ancillary court documents and defendants’ statements, which we assigned to the five categories (profit/ commercial gain; thrill/ sport; necessity for obtaining food or protecting livestock/ crops; antipathy towards governmental and law enforcement bodies; and traditional/ cultural reasons) proposed by Nurse [14]. We also recorded offender nationality.

Corresponding to CL criteria, we categorized crime seriousness as minor offence with probation or an administrative fine; moderate offence with < 5 years fixed-term imprisonment; serious offence with 5–10 years of fixed-term imprisonment; and very serious offence with 10–15 years of fixed-term imprisonment. When the case was (re-)tried on multiple occasions, the final judgement was used to allocate crime seriousness. It was not possible to link separate prosecutions for recidivists because offender anonymity precluded cross-referencing records.

### Species persecuted

We tested for any relationship between perpetrator socio-demographic profile and taxon, focusing primarily on ten frequently persecuted families: Accipitridae (raptors, Old World vultures), Strigidae (owls), Phasianidae (pheasants), Cercopithecidae (Old World monkeys), Bovidae (e.g. gaur, antelopes, serow, takin), Elephantidae (African/ Asiatic elephants), Rhinocerotidae (rhinos), Felidae (cats), Ursidae (bears, giant panda) and Manidae (pangolins).

### Statistical analysis

The explanatory variables sex, age, schooling and occupation were not independent. Schooling and occupation had the highest contingency coefficients (*C* = 0.364) for explaining crime seriousness and type of illegal activity. We thus tested how socio-demographic factors related to crime seriousness using ordinal logistic regression, type of illegal activity using multinomial logistic regression, via odds ratio (OR) prediction, and taxon involved using chi-square goodness-of-fit tests. We calculated standardized residuals (std. res) if chi-square tests were significant to determine which demographic factors were influential [46]. Chi-square goodness of fit was also used to test socio-demographics in relation to crimes with a ‘traditional/ cultural’ motivation; while sample size dictated an OR approach to contrast offender profiles motivated by tradition/ culture against protecting livestock/ crop. Statistical analyses were performed using the Statistical Package for the Social Sciences (SPSS) software (IBM, 2011).

## Results

### Offender socio-demographic profiles

**Fig 1:**
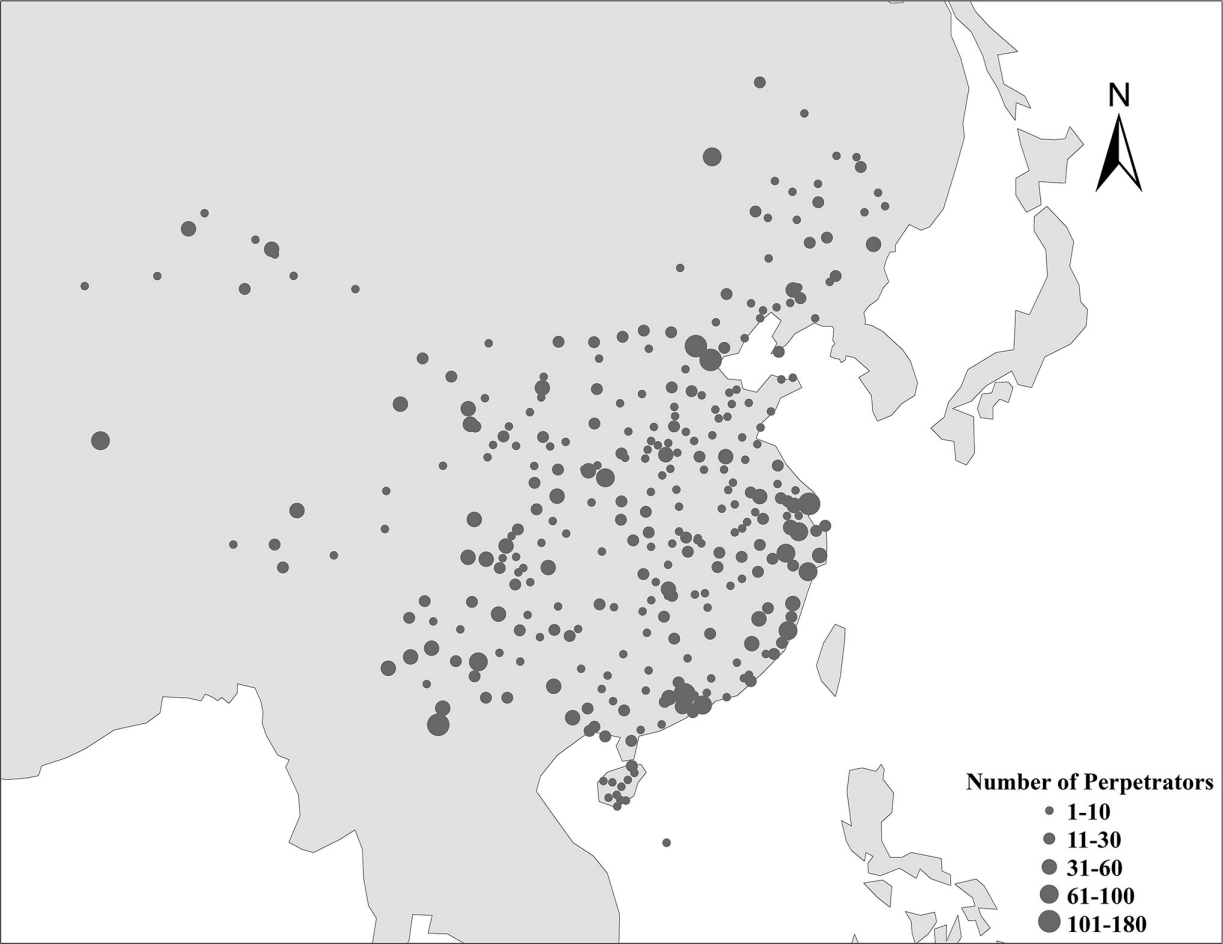
Number and geographical distribution of perpetrators in mainland China. Dots represent the city local to where wildlife crimes were prosecuted. The base map was created with Natural Earth Dataset(http://www.naturalearthdata.com/).

We examined 4,735 cases involving 7,244 wildlife offenders from 5,509 judgement documents. 99.5% (7,206) convictions involved Chinese citizens representing 310 (of 338) mainland China prefectures (Fig 1). 38 (0.5%) foreign nationals were imprisoned, including offenders from Egypt (3 individuals), Japan (1), Laos (1), Mongolia (3), Myanmar (7), Niger (1), Nepal (2), North Korea (2), Pakistan (1), Russia (4), South Korea (1) and Vietnam (4); the nationalities of 8 other foreign perpetrators were unspecified.

Average offender age was c. 40 years (SD = 11 years: males, mean = 39.54, SD = 11.40, n = 4,305; females, mean = 39.24, SD = 11.34, n = 393) and the 30–44 age group accounted for the majority (43.9%) of wildlife crimes. 93.0% (6,644) of offenders were male. 9.4% (462) were never schooled; 30.9% (1,524) of offenders had only primary school education; the remaining 59.7% (2,943) completed compulsory education up to junior middle school, age ca. 15 (38.3%, 1,889), senior middle school, age ca. 18 (12.9%, 637), or college and higher education (8.4%, 414). Of 3,934 offenders, for whom occupation was recorded, 48.4% (1,905) were agricultural workers, 36.2% (1,426) were non-agricultural workers and 15.3% (602) were unemployed.

### Crime seriousness

Among 3,108 offenders with full explanatory variables, 1847 (59.4%) committed minor, 845 (27.2%) moderate, 290 (9.3%) serious and 126 (4.1%) very serious offences.

Men had a higher probability of committing more serious crime categories than women (β = 0.70, *P* < 0.05). Younger age-class offenders aged 15–29 (β = 0.73, *P* < 0.05) and 30–44 (β = 0.71, *P* < 0.05) were much more likely to commit crimes than the > 60 age group; especially serious crime (Table 1). Less educated offenders were likely to commit more serious crimes, especially unschooled offenders compared to those with college and higher education (β = 1.10, *P* < 0.05). Workers in the agricultural (β = 0.19, *P* < 0.05) and unemployed (β = 0.30, *P* < 0.05) categories were more likely to commit serious crimes than those in the non-agricultural category.

**Table 1:** Ordinal logistic regression showing relationships between explanatory socio-demographic variables and crime seriousness (n = 3,108).

	Estimate *β*	*P*	95% Wald confidence interval	
**(Intercept)**				
**Minor offence**	2.38	<0.05	1.84–2.93	
**Relatively minor offence**	4.31	<0.05	3.72–4.91	
**Serious offence**	9.78	<0.05	8.34–11.22	
**(Threshold)**				
**Gender**				
** Male**	0.70	<0.05	0.39–1.01	
** Female**	0	-	-	
**Age**				
** 15–29**	0.73	<0.05	0.37–1.09	
** 30–44**	0.71	<0.05	0.37–1.06	
** 45–59**	0.48	<0.05	0.13–0.821	
** 60-**	0	-	-	
**Schooling**				
** No schooling**	1.10	<0.05	0.74–1.47	
** Primary school**	0.80	<0.05	0.48–1.12	
** Junior middle school**	0.64	<0.05	0.33–0.95	
** Senior secondary school**	0.39	<0.05	0.04–0.74	
** College and higher level**	0	-	-	
**Occupation**				
** Agricultural workers**	0.19	<0.05	0.05–0.34	
** Unemployed**	0.30	<0.05	0.11–0.49	
** Nonagricultural workers**	0	-	-	

Log likelihood ratio chi squares test = 125.61, *P* < 0.05, Hosmer–Lemeshow goodness of fit test (Chi-square 269.78; *P* = 0.76).

### Type of illegal activity

Among 3,108 offenders with full explanatory variables, 1,010 (32.5%) committed hunting crimes, 108 (3.5%) were prosecuted for smuggling, and 1,990 (64.0%) offenders exclusively perpetrated crimes involving domestic trafficking.

Against the benchmark of domestic trafficking, men were 5.76 times more likely than women to commit hunting crimes (OR = 5.76, 95% CI = 3.58–9.26) and 1.46 times more likely to commit smuggling (OR = 1.46, 95% CI = 0.72–2.96); hunting offences were predominantly committed by those in the agricultural worker category (OR = 2.75, 95% CI = 2.26–3.35) and involved offenders that did not attend senior middle school; who were also least likely to be involved in smuggling (Table 2).

**Table 2:** Estimate *β* (95% Wald confidence interval) from multinomial logistic regression comparing socio-demographic explanatory variables relating smuggling and hunting to domestic trafficking.

	Smuggling	Hunting
**Sex**		
** Male**	1.46 (0.72–2.96)*	5.76 (3.58–9.26)*
** Female**		.
**Age**		
** 15–29**	1.39 (0.31–6.12)	0.94 (0.63–1.40)
** 30–44**	1,81 (0.43–7.69)	0.91 (0.63–1.31)
** 45–59**	1.57 (0.36–6.83)	0.98 (0.67–1.41)
** 60-**		.
**Schooling**		
** No schooling**	0.28 (0.08–0.98)*	8.25 (4.77–14.29)*
** Primary school**	0.42 (0.22–0.81)*	6.19 (3.74–10.26)*
** Junior middle school**	0.54 (0.31–0.92)*	3.34 (2.03–5.48)*
** Senior middle school**	0.51 (0.27–0,98)*	1.68 (0.96–2.93)
** College and higher level**	.	.
**Occupation**		
** Agricultural workers**	0.69 (0.41–1.14)	2.75 (2.26–3.35)*
** Unemployed**	1.26 (0.78–2.06)	1.04 (0.78–1.38)
** Non-agricultural workers**	.	.

*Significance at 0.05 level.

### Taxon involved

Of 3,856 offenders, 510 committed crimes involving multiple taxonomic families (414 offended against 2 families, 81 against 3 families, 7 against 4 families, 1 against 5 families, 7 against 6 families). These were counted separately against each family in Table 3.

**Table 3:** Chi-square goodness-of-fit test of offender socio-demographic characteristics and type of crime activity categories in relation to taxa.

	Total	Accipitridae (n = 513)	Bovidae (n = 590)	Cercopithecidae (n = 290)	Elephantidae (n = 898)	Felidae (n = 276)	Manidae (n = 543)	Phasianidae (n = 465)	Rhinocerotidae (n = 102)	Strigidae (n = 376)	Ursidae (n = 402)
**Sex**					P<0.05				P<0.05		
** Male**	93.0%	93.8% (0.1)	95.6% (0.3)	86.4% (-0.7)	84.5% (-0.9)	95.3% (0.2)	92.6% (0.0)	94.8% (0.2)	83.8% (-1.0)	89.6% (-0.4)	94.5% (0.2)
** Female**	7.0%	6.2% (-0.3)	4.4% (-1.0)	13.6% (2.5)	15.5% (3.2)	4.7% (-0.9)	7.4% (0.2)	5.2% (-0.7)	16.2% (3.5)	10.4% (1.3)	5.5% (-0.6)
**Age**					P<0.05				P<0.05	P<0.05	P<0.05
** 15–29**	22.4%	29.9% (1.6)	15.5% (-1.5)	31.1% (1.8)	29.6% (1.5)	21.8% (-0.1)	16.0% (-1.4)	18.5% (-0.8)	33.3% (2.3)	12.7% (-2.0)	12.8% (-2.0)
** 30–44**	43.9%	40.2% (-0.6)	44.4% (0.1)	38.6% (-0.8)	49.6% (0.9)	40.7% (-0.5)	48.0% (0.6)	38.4% (-0.8)	47.0% (0.5)	34.3% (-1.4)	49.4% (0.8)
** 45–59**	28.7%	22.4% (-1.2)	36.4% (1.4)	23.7% (-0.9)	18.4% (-1.9)	32.9% (0.8)	33.2% (0.8)	36.1% (1.4)	19.7% (-1.7)	38.8% (1.9)	35.0% (1.2)
** 60-**	5.0%	7.5% (1.1)	3.7% (-0.6)	6.6% (0.7)	2.4% (-1.2)	4.6% (-0.2)	2.8% (-1.0)	6.9% (0.8)	0.0% (-2.2)	14.2% (4.1)	2.8% (-1.0)
**Schooling**			P<0.05		P<0.05		P<0.05	P<0.05	P<0.05		
** None school**	9.4%	7.3% (-0.7)	17.3% (2.6)	8.7% (-0.2)	2.4% (-2.3)	8.5% (-0.3)	4.6% (-1.6)	14.2% (1.6)	1.8% (-2.5)	12.0% (0.8)	9.2% (-0.1)
** Primary school**	30.9%	33.6% (0.5)	40.0% (1.6)	32.5% (0.3)	13.7% (-3.1)	23.5% (-1.3)	26.9% (-0.7)	44.4% (2.4)	21.1% (-1.8)	38.6% (1.4)	28.9% (-0. 4)
** Junior middle school**	38.3%	40.6% (0.4)	29.6% (-1.4)	33.5% (-0.8)	37.8% (-0.1)	39.9% (0.3)	51.4% (2.1)	31.0% (-1.2)	36.8% (-0.2)	38.6% (0.0)	45.1% (1.1)
** Senior middle school**	12.9%	11.8% (-0.3)	8.3% (-1.3)	16.0% (0.9)	22.5% (2.7)	14.6% (0.5)	13.0% (0.0)	7.0% (-1.6)	24.6% (3.3)	7.3% (-1.6)	11.3% (-0.4)
** College and higher level**	8.4%	6.7% (-0.6)	4.7% (-1.3)	9.2% (0.3)	23.6% (5.2)	13.6% (1.8)	4.1% (-1.5)	3.4% (-1.7)	15.8% (2.6)	3.5% (-1.7)	5.6% (-1.0)
**Occupation**			P<0.05	P<0.05	P<0.05	P<0.05		P<0.05	P<0.05	P<0.05	
** Agricultural workers**	48.4%	55.7% (1.0)	64.6% (2.3)	48.6% (0.0)	21.4% (-3.9)	34.8% (-2.0)	47.0% (-0.2)	74.1% (3.7)	23.4% (-3.6)	70.0% (3.1)	44.5% (-0.6)
** Unemployed**	15.3%	11.4% (-1.0)	11.4% (-1.0)	23.7% (2.1)	21.0% (1.5)	27.1% (3.0)	12.9% (-0.6)	6.5% (-2.2)	9.4% (-1.5)	8.5% (-1.7)	19.8% (1.2)
** Nonagricultural workers**	36.3%	32.9% (-0.6)	24.0% (-2.0)	27.7% (-1.4)	57.6% (3.5)	38.1% (0.3)	40.2% (0.6)	19.4% (-2.8)	67.2% (5.1)	21.6% (-2.4)	35.7% (-0.1)
**Activity**			P<0.05	P<0.05	P<0.05	P<0.05	P<0.05	P<0.05	P<0.05	P<0.05	P<0.05
** Smuggling**	5.5%	0.0% (-2.3)	2.9% (-1.1)	0.3% (-2.2)	14.1% (3.7)	9.8% (1.8)	11.5% (2.6)	0.2% (-2.3)	19.6% (6.0)	0.0% (-2.3)	7.2% (0.7)
** Hunting**	32.4%	37.8% (0.9)	55.1% (4.0)	22.4% (-1.8)	0.3% (-5.6)	13.4% (-3.3)	7.6% (-4.4)	56.3% (4.2)	0.0% (-5.7)	50.0% (3.1)	19.2% (-2. 3)
** Domestic trafficking**	62.1%	62.2% (0.0)	42.0% (-2.6)	77.2% (1.9)	85.5% (3.0)	76.8% (1.9)	80.9% (2.4)	43.4% (-2.4)	80.4% (2.3)	50.0% (-1.5)	73.6% (1.5)

Standardized residuals in brackets.

Offences relating to Elephantidae (83.9%), Manidae (68.5%) and Rhinocerotidae (77.2%) predominantly involved offenders with at least a junior middle school education; these taxa were most often smuggled (versus benchmark of all 7.206 native offenders: std. res = 3.7; 2.6 and 6.0, respectively; Table 3). Interestingly, women were over-represented as offenders in elephant and rhinoceros smuggling/trafficking crimes (linked to ivory and horn) versus general levels of offending (std. res = 3.2 and 3.5 respectively); offenders were predominantly aged 15–29 (std. res = 1.5 and 2.3 respectively) and mostly from the non-agricultural worker category (std. res = 3.5 and 5.1 respectively).

Offences relating to Bovidae (std. res = 4.0), Phasianidae (std. res = 4.2) and Strigidae (std. res = 3.1) mainly involved hunting (Table 3) using illegal firearms in 117, 108 and 28 cases respectively (incurring parallel prosecution). These offences were perpetrated mostly by the agricultural worker category (std. res = 2.3, 3.7 and 3.1 respectively), with below junior middle school education. Notably, Strigidae crimes were perpetrated predominantly by older offenders (age group 60 and above: std. res = 4.1).

Compared to overall offence patterns, crimes involving Cercopithecidae (std. res = 1.9), Felidae (std. res = 1.9) and Ursidae (std. res = 1.5) mainly involved domestic trafficking (Table 3). The unemployed perpetrated the majority of crimes against Cercopithecidae and Felidae (std. res = 2.1 and 3.0 respectively). Deviating from general patterns, crimes involving Ursidae were predominantly perpetrated by the 45–59 age group (std. res = 1.2). Crimes involving Accipitridae conformed with overall offender profile patterns (Table 3).

### Offender motivation

Within the sub-sample of offenders for which we could deduce probable motivation (n = 489), 201 offenders were prosecuted for domestic trafficking, and 288 for hunting (see below). Of the 201 domestic trafficking offenders, 45.8% were motivated by the intention to trade in pets/working animals (e.g. Northern goshawk (*Accipiter gentilis*) and macaques (*Macaca* spp.)); 25.4% were trading in food species (e.g. pangolin (*Manis* spp.), silver pheasant (*Lophura nycthemera*), Asiatic black bear (*Ursus thibetanus*), wild yak (*Bos mutus*)); 16.4% were involved in Chinese Traditional Medicine (CTM; e.g. pangolin scales, monkey skeletons, tiger bone/ penis); 5.0% in gifts (e.g. ivory, rhino horn, elongated tortoise (*Indotestudo elongata*), pangolin); 4.0% in ornaments (e.g. mounted deer heads, eagle claws, rhino horn); 2.5% in collections (e.g. bear fur, lion canine, ivory); and 0.9% were motivated by the use of animals in religious practices / exorcisms (e.g. Crested goshawk (*Accipiter trivirgatus*), Collared owlet (*Glaucidium brodiei*)) (Table 4).

**Table 4:** Contrasting hunter and consumer motives recorded in the judgement documents for ten principal taxa. Motivations were inferred from court and/or the offenders’ statements, and assigned to the categories proposed by Nurse [14].

Motives [14]	Hunters	Consumers
**Tradition and cultural reasons**	Food for personal use ^A^^,^ ^B^^,^ ^C^^,^ ^F^^,^ ^P^^,^ ^S^^,^ ^U^ Gift to employer ^S^ Pet for pleasure ^A^^,^ ^C^^,^ ^P^^,^ ^S^ CTM for rheumatism, mental illness or health supplement ^A^^,^ ^B^^,^ ^C^^,^ ^S^	Collecting and possessing products with high commercial value ^A^^,^ ^E^^,^ ^M^^,^ ^U^ Ornaments and jewelry ^A^^,^ ^F^^,^ ^P^^,^ ^R^ Pets for pleasure or as working animals ^A^^,^ ^C^^,^ ^M^^,^ ^P^^,^ ^S^^,^ ^U^ Food for private consumption ^A^^,^ ^B^^,^ ^C^^,^ ^M^^,^ ^P^^,^ ^S^^,^ ^U^ Gifts for friends or family members ^B^^,^ ^E^^,^ ^M^^,^ ^P^^,^ ^R^ Religion or apotropaic spiritualism, to ward off evil spirits, or Buddhist wildlife release ^A^^,^ ^S^ CTM for rheumatism, mental illness, dizziness, bronchitis, headache, gastrosis, cough, dermatosis, hepatopathy, or health supplement ^A^^,^ ^B^^,^ ^C^^,^ ^F^^,^ ^M^^,^ ^P^^,^ ^S^^,^ ^U^
**To obtain subsistence food or protect livestock/ crops**	Retribution killing of wild animals that damage crops and/ or endanger livestock or human life ^A^^,^ ^E^^,^ ^F^^,^ ^P^^,^ ^S^^,^ ^U^	
**Commercial profit**	Extra income ^A^^,^ ^B^^,^ ^C^^,^ ^P^^,^ ^S^^,^ ^U^	

^A^ Accipitridae

^B^ Bovidae

^C^ Cercopithecidae

^E^ Elephantidae

^F^ Felidae

^M^ Manidae

^P^ Phasianidae

^R^ Rhinocerotidae

^S^ Strigidae

^U^ Ursidae.

Of 288 hunters prosecuted for traditional/ cultural hunting, the motivation of 70.4% was to acquire food (e.g. Chinese goral (*Naemorhedus griseus*), Chinese serow (*Capricornis milneedwardsii*), takin *(Budorcas taxicolor*), Siberian ibex (*Capra sibirica*), Lady Amherst's pheasant (*Chrysolophus amherstiae*)), 17.3% aimed to trap exotic pets (e.g. Northern goshawk, rhesus Monkey (*Macaca mulatta*), Eurasian sparrowhawk (*Accipiter nisus*), golden eagle (*Aquila chrysaetos*), Eurasian eagle-owl (*Bubo bubo*)); 3.8% for CTM (e.g. Milne-Edwards’ macaque (*Macaca thibetana*), Eurasian buzzard (*Buteo buteo*), takin); additionally 7.3% of these hunting offenders were motivated by their perceived need to protect livestock/ crops from conflicts with wildlife.

Traditional/ cultural hunters were most likely to belong to the agricultural worker category (std. res = 5.0) with below junior middle school education (no schooling: std. res = 3.4; primary school educated: std. res = 3.5). Hunting crimes motivated by livestock/ crop protection involved older than hunters than hunting motivated by traditional/ cultural reasons (45–59 age group: 43.8% vs 25.9%; 60 and over: 37.5% vs 7.8%).

## Discussion

We show that wildlife crime is prosecuted rigorously across China, where China Judgements Online provided a comprehensive and accessible resource detailing wildlife offenders. As with most data on illegal wildlife trade [47], some details were incomplete, and few judgement cases prosecuted non-CITES species/ species not listed on LFSSP. Nevertheless, we clearly detected that age, sex, education and occupation were robust predictors of the propensity to offend against wildlife in China. This corroborates studies profiling crime in general, both in Eastern and Western societies [24,48,49], although while wildlife offenders, especially those committing serious crimes, followed a typical socio-demographic criminal profile [22,50], we identified significant biases relating to taxa involved, type of illegal activity, and offender motivation.

### Sex and age

Men offended at much higher rates than women, especially committing more serious wildlife crimes, mirroring patterns in all other crime categories except prostitution [22,31,51]. Wildlife criminals in China were similar to the general criminals in Chinese society, but generally older (late 20s, or older) than the typical age profile of general offenders in the US (general Western pattern; late teens) [49]. We caution, however, of the risk of sweeping assumptions: while Sollund [32] attributes a male bias in wildlife offending to hegemonic masculinity, this is less likely to be the basis of the bias in China, where the idealized model of masculinity is differently connected with the concepts of *wen* (cultural attainment) and *wu* (martial valour) [52–54]. Particularly the act of hunting by predominantly those working in the agricultural category, is much more pragmatic in China, to put food on the family table, or alleviate pressures on household budgets through the small-scale sale of poached bushmeat [55]; subsistence rather than pursuit of trophies and kudos–although we acknowledge the occurrence of some recreational hunting in China [56,57].

Examining CTM, the reason that this activity is perpetrated particularly by older offenders may be due to a stronger traditional belief in its efficacy, or a personal reliance on these remedies for ailments [58]. For example, in 2018, a 73-year-old male peasant with junior middle schooling, was sentenced to 16 years in jail in Guangxi Province for illegally hunting 19 greater coucals (*Centropus sinensis*; a type of cuckoo species of least concern on the IUCN Red List of Threatened Species, but protected under China’s LFSSP) using noose traps near his home and selling them at a rural market as CTM remedy for rheumatism.

### More extensive schooling associated with trafficking/ smuggling

A Swedish study found that one additional year of schooling decreased male conviction likelihood by 6.7% and incarceration by 15.5% [48]. Our data, however, indicated the opposite effect for ‘skilled’ wildlife crime categories, such as smuggling. The majority of ivory [44,59], pangolin [2] and rhino horn [40] trade originates outside of China, therefore smugglers require better management, organization, internet and/ or computer skills (associated with higher education) than do agricultural hunters [60]. These skills enable offenders with higher schooling to negotiate illicit international trade deals, while attempting to evade detection [61,62]. For example, 11 offenders (9 men, 2 women), comprising a network illegally trafficking a tiger pelt, 1.35 kg of rhino horn and 32.55 kg of ivory from Vietnam, were sentenced to 8 years in jail in 2016. Five of these offenders were college educated; the others had finished senior middle school.

### Agricultural workers and hunting

Agricultural workers often have ready means and opportunity to hunt [44], therefore the motivation behind poaching may largely be opportunistic intended to aide subsistence, with a relatively small proportion intended for commercial profit [63].

Private possession of firearms is strictly prohibited in China; therefore the hunters involved in those 279 shooting-related offences that detailed firearm type (from a total of 487) improvised and used a variety of unlicensed air guns (24.7%), enhanced nail guns (23.7%) and simple homemade flint-lock muskets (51.6%). These low muzzle-velocity guns risk wounding animals, causing considerable suffering. For example, in 2015, a 37-year-old male agricultural worker from Yunnan Province (with only primary schooling), was sentenced to 12 years imprisonment for hunting a giant panda (*Ailuropoda melanoleuca*) near his residence with a homemade flintlock musket and selling its body parts as Asian black bear. He claimed his actions were retribution for the panda killing one of his sheep. He received an additional 3-year sentence for possessing an illegal firearm. Also in 2015, a 41-year-old male agricultural worker from Yunnan Province (no schooling) was sentenced to 10 years, for illegally killing an Asian elephant (*Elephas maximus*) with a homemade flintlock musket, where the offender claimed the elephant was doing harm to his rubber plantation.

New technologies continually enhance hunting efficiency. For instance, a 46-year-old male agricultural worker from Shaanxi Province (primary school education) was sentenced to 10 years in 2014 for accidentally killing a leopard (*Panthera pardus*) with an electrocution trap comprising a truck battery and a voltage transformer connected to insulating rods at a height of 0.2–0.5 m, spanned with 300m of fencing wire. His intended quarry was wild boar, Siberian roe deer, badgers and hares, but his trap was indiscriminate and also posed a threat to people.

### Understanding motivations

We see an emergent pattern where certain wildlife offences, especially trading and smuggling, are motivated by commercial gain, others by traditional/ cultural reasons, and others by pragmatism or necessity, such as obtaining free food or protecting livestock/ crops [64]. These offender motivations were thus reflected in the taxa involved. 25.3% of hunting crimes involved species consumed as a subsistence food source, and involved species widely distributed in China, mostly wild Bovidae (e.g., Chinese goral (*Naemorhedus griseus*), Chinese Serow (*Capricornis milneedwardsii*), blue sheep (*Pseudois nayaur*)), Phasianidae and Strigidae. These species were hunted opportunistically, predominantly by workers in the agricultural category, with less schooling, reflecting socio-economic profiles of subsistence hunters elsewhere [65,66]. In contrast, in the United States [67] and Sweden [68], most (illegal) hunting is for recreation or trophies, involving better educated and more affluent individuals [69]. In Russia, hunting species outside of the law is often perpetrated by the social elite, immune from prosecution [70], again to demonstrate social status. Our assessment was that elitism is not a factor in China. In China, we perceive that that proportion of wildlife crime committed by the hunters is driven more by necessity than by any tendency toward anti-authoritarian deviancy (which is often again linked to machismo) [71].

Domestic trafficking offences involved mostly Cercopithecidae, Felidae and Ursidae, including a substantial proportion of animals bred illegally in captivity. For example, a 53-year-old male offender in Guangdong Province (junior middle schooling), who operated a restaurant, was sentenced to 12 years in jail in 2017, for illegally trafficking expensive wild food delicacies, including tiger (*Panthera tigris*, c. 17kg meat, 17 claws), Asiatic black bear (*Ursus thibetanus*, c. 4kg meat) and Nicobar crab-eating macaque (*Macaca fascicularis*, c. 3kg meat). A licensed breeding facility rejected the offender’s accusations that they had supplied this meat on this and previous occasions; instead contending that they had ‘lost’ the carcasses of dead animals in 2016.

Demand for luxury goods and food is a major driver of global wildlife trade (cited in 35% of 374 reports), followed by traditional medicine (25% of reports) and pets (22%) [72]. As prosperity increases in China, animal products (ivory, rhino horn, big cat products, etc) [73], exotic pets [41] and even animal ingredients in CTM, are perceived as conferring cachet upon their owners [2,43]. The judgement documents we examined did not provide enough information to formally analyze for any relationship between offender socio-demographics and propensity to trade in luxury goods, but, interestingly, women were more highly represented in offences involving rhino horn and elephants/ ivory than in any other type of wildlife crime–often involving artisan or retail work rather than other more physically demanding/ violent wildlife crimes.

Recidivism and involvement in other kinds of crime were also implicit factors in our dataset. For instance, in 2018, a 33-year-old man from Fujian Province, with a prior criminal record for rape and larceny, was sentenced to 5 years for trading and collecting animal products via e-commerce (Taobao.com) and social media (WeChat) platforms. His collection included an Asiatic black bear skin and gall bladder, a stuffed grey wolf (*Canis lupus*), a Reeves's pheasant (*Syrmaticus reevesii*), a crested serpent-eagle (*Spilornis cheela*), an Eurasian buzzard (*Buteo buteo*), two golden pheasants (*Chrysolophus pictus*), the horn of a Saiga (*Saiga tatarica*) and two lion (*Panthera leo*) canine teeth.

Aside from the exploitation of wild animals for profit or food, 21 offenders were involved in illegal interventions intended to deter or kill animals that caused real or perceived commercial losses due to human-wildlife conflict [74,75]. For instance, in 2018, a 26-year-old male herdsman in Tibet (junior middle schooling), was sentenced to 5 years in jail (subsequently exempted from criminal liability upon appeal), for illegally hunting a snow leopard (*Panthera uncia*) with a coil-spring leg-hold trap. His defense was that leopards were killing dozens of his sheep each year, and he had not been offered any state compensation.

According to the definition of criminal groups given by Wyatt et al. [60], only 0.9% of offences were committed by corporate or organized crime. Nevertheless, the scale and international reach of these cartels has scope to significantly impact national and global biodiversity [76]: for example, between 2013–2014 an unlicensed company in Guangxi Province was prosecuted for smuggling 4,195 pangolins from Vietnam. Nine men and one woman (ages spanning 20–51 years; all with no more than secondary schooling) were charged; 9 suspects escaped. Three principle company managers, involved in receiving and distributing goods, were sentenced to 10 years; seven others were sentenced to 1–6 years, as accessories.

## Conclusions

Numerous studies have dealt extensively with how wildlife crime and trade might be tackled by educating and reforming consumer practices [10]. In terms of sanction, threats of punishment and incarceration provide a general deterrent against offending [23,77,78], weighed against the likelihood of detection and successful prosecution [41]. In this sense, the per capita rates of prosecution we report seem rather low, and there is other evidence that minor illegal wildlife trading infringements are not always pursued in China; for example, as evidenced by our own work investigating illegal trade in parrots and turtles [41,79] and the illegal sale of protected species through wet markets [80].

Wildlife crime in China may therefore be better mitigated through incentive-based interventions [81]. Many such generic strategies are well-established, such as providing education on welfare and the impacts of biodiversity loss, or increasing stake-holder involvement in the management of wildlife [6,82,83], but through our socio-demographic analysis we advocate here some more specific intervention strategies targeted at the primary constituencies involved in wildlife crime in Chinese society [84,85]. Herein the type of the Chinese agricultural system must be taken into account, where individuals work their own small-holdings, and thus have a sense of custodianship, transcending each rural farming community. Without a victim coming forward to report that they have suffered theft or assault, wildlife crime can be hard to detect [25]. Therefore, citizen reporting can be a vital policing tool [86]. Of course, ability to conceal wildlife crime, or complicitness in failing to report on others is also a function of whether a few cuckoos are being trapped quietly, or an elephant is being shot with a musket. Within rural communities, no one will easily report their friends and neighbors; however this community ethic can be put to use as a device for making wildlife crime socially unacceptable, where, equally, no one wants to be a pariah. The Chinese Confuscion, Tao and Buddhist tradition is thus important, because it values the natural harmony between man’s ritual propriety and the natural principles of the universe [87,88]. Thus, leveraging wildlife exploitation as a source of bad karma, counter to the ethic of feeling an emotional engagement with the land and nature implicit to these religious philosophies (especially Taoism), presents a potential opportunity to reduce criminal wildlife exploitation at the community level.

Our analysis leads us to advocate a complementary series of strategies to redress offences that engage with Chinese society [84]:

1. To target public information messaging toward men involved in agricultural work and to provide economic incentives to protect wildlife. The former might be achieved through the use of slogans painted on buildings in rural areas and local cable broadcasts. Messaging should be simple and clear, ideally connecting with long-standing moral traditions that emphasize and extol the preservation of wildlife and nature. The latter could build on their role as custodians of the land and pay them benefits for protecting wildlife and habitats.
2. To diversify policing approaches and resources, not only to patrol and respond to rural crime and market trade, but to target more sophisticated trafficking and smuggling crimes perpetrated by better educated offenders, skilled at evading detection.
3. To tackle poverty and indigence through providing alternative and/or better paid jobs in rural communities to alleviate need–based animal exploitation [89] and to improve compensation schemes and access to these schemes (in relation to literacy) for those farmers suffering crop damage or livestock predation [90].
4. To promote non-CTM treatments among the elderly as a way to reduce their dependence on wildlife remedies [91].
5. Crimes of greed, rather than need, and especially those involving organized crime, should be prosecuted within a wider framework of disbanding (especially international) crime syndicates and rehabilitating career criminals [92].

Ultimately, a clearer understanding of offender typology and motivation may enable legislative reform, informing laws and rehabilitation that better protect wildlife in China and internationally.
